# Cell Architecture of the Giant Sulfur Bacterium *Achromatium oxaliferum*: Extra-cytoplasmic Localization of Calcium Carbonate Bodies

**DOI:** 10.1093/femsec/fiz200

**Published:** 2019-12-24

**Authors:** Sina Schorn, Verena Salman-Carvalho, Sten Littmann, Danny Ionescu, Hans-Peter Grossart, Heribert Cypionka

**Affiliations:** 1 Max Planck Institute for Marine Microbiology, Celsiusstraße 1, 28359 Bremen, Bremen, Germany; 2 Institute for Chemistry and Biology of the Marine Environment, Carl-von-Ossietzky University of Oldenburg, Carl-von-Ossietzky Straße 911, 26133 Oldenburg, Oldenburg, Germany; 3 Leibniz-Institute of Freshwater Ecology and Inland Fisheries, Alte Fischerhütte 2, 16775 Stechlin, Berlin, Germany; 4 Institute of Biochemistry and Biology, Potsdam University, Karl-Liebknecht Straße 24-25, 14476 Potsdam, Potsdam, Germany

**Keywords:** Sulfur-bacteria, calcium carbonate inclusions, extra-cytoplasmic pockets, calcite

## Abstract

*Achromatium oxaliferum* is a large sulfur bacterium easily recognized by large intracellular calcium carbonate bodies. Although these bodies often fill major parts of the cells’ volume, their role and specific intracellular location are unclear. In this study, we used various microscopy and staining techniques to identify the cell compartment harboring the calcium carbonate bodies. We observed that *Achromatium* cells often lost their calcium carbonate bodies, either naturally or induced by treatments with diluted acids, ethanol, sodium bicarbonate and UV radiation which did not visibly affect the overall shape and motility of the cells (except for UV radiation). The water-soluble fluorescent dye fluorescein easily diffused into empty cavities remaining after calcium carbonate loss. Membranes (stained with Nile Red) formed a network stretching throughout the cell and surrounding empty or filled calcium carbonate cavities. The cytoplasm (stained with FITC and SYBR Green for nucleic acids) appeared highly condensed and showed spots of dissolved Ca^2+^ (stained with Fura-2). From our observations, we conclude that the calcium carbonate bodies are located in the periplasm, in extra-cytoplasmic pockets of the cytoplasmic membrane and are thus kept separate from the cell's cytoplasm. This periplasmic localization of the carbonate bodies might explain their dynamic formation and release upon environmental changes.

## Teaser

Visualization of the unique cell architecture of *Achromatium oxaliferum*, a bacterium of exceptionally large size and unique morphology.

## INTRODUCTION

Bacteria of the genus *Achromatium* are found in oxic-anoxic transition zones of sediments worldwide, including freshwater (Schewiakoff 1893; Babenzien 1991; Head et al. 1996; Glöckner et al. 1999; Gray et al. 1999, Schorn and Cypionka 2018), brackish (Mansor et al. 2015) and marine environments (Salman et al. 2015). They belong to the large sulfur-oxidizing bacteria, and are easily recognized microscopically due to the calcium carbonate bodies deposited inside their cells, and smaller sulfur globules (Babenzien 1991; Head et al. 2000b). While the occurrence of intracellular sulfur globules is common among large sulfur bacteria (Larkin and Henk 1996; Schulz et al. 1999) the calcium carbonate bodies are a unique feature of the genus *Achromatium*. Although these bodies may fill up more than 70% of *Achromatium*‘s cell volume (Head et al. 2000b), their biological role is still under debate (Salman et al. 2015, and detailed review in Gray 2006). It is assumed that the calcium carbonate bodies in *Achromatium* influence the buoyancy of the cells (Babenzien 1991). The increase in weight by calcium carbonate incorporation into the cell body could anchor the cells in the sediment and thereby prevent them from being suspended into the water column. Calcium carbonate was also suggested to buffer intracellular pH fluctuations during sulfur oxidation (La Rivière and Schmidt 1981; Gray 2006; Salman et al. 2015). This would require a cytoplasmic localization of the carbonate. In recent studies it was found that the calcium carbonate content of the cell changes with environmental conditions, and may be dependent on the sulfide concentration (Salman et al. 2015) or redox conditions (Yang et al. 2019). This suggests that the cells have the ability to quickly adjust their cellular calcium carbonate content.

Compartmentalization of the cell and deposition of storage compounds is a common trait among large sulfur bacteria. For example, among members of the *Beggiatoaceae* most of the cell volume is occupied by a large, central, membrane-surrounded vacuole that is used for nitrate storage (Schulz and Jørgensen 2001). Among the purple sulfur bacteria, *Chromatiaceae* are the closest relatives of *Achromatium* and their sulfur globules appear to be located in the periplasmic space as previously shown by genetic analyses (Pattaragulwanit et al. 1998). In single cells of both *Beggiatoaceae* and *Achromatium*, hundreds to thousands of DNA spots have been found (Lane and Martin 2010; Salman et al. 2015; Salman-Carvalho et al. 2016, Ionescu et al. 2017). Recently, we detected that single *Achromatium oxaliferum* cells harbor a genetic diversity which is generally typical of whole bacterial communities (Ionescu et al. 2017). The presence of a membrane-surrounded vacuole inside *Achromatium*, however, has so far not been reported. Also, whether the calcium carbonate bodies are located within the cytoplasm, in separate compartments, or in the periplasm, is currently unknown. Yet, they were found to be surrounded by membranes (Head et al. 2000b), which indicated that the cell harbors them in a separate compartment. Knowing the cellular location of the calcium carbonate bodies is not only fundamental for understanding the cell architecture of *Achromatium*, in particular the cell's compartmentalization, but it could also provide information on the physiological role of calcium carbonate bodies, possibly explain the previously observed dynamics of calcium carbonate precipitation and dissolution (Salman et al. 2015; Yang et al. 2019), and shed light on their contribution to the large intracellular genetic diversity of individual *Achromatium* cells (Ionescu et al. 2017).

In the present study, we have used various microscopy techniques including bright-field, fluorescence, confocal, superresolution and scanning electron microscopy to analyze the cell architecture of *Achromatium oxaliferum*. We visualized slime capsules around the cells, internal membrane structures, cytoplasm, DNA distribution and the calcium carbonate-harboring cavities. Our findings give evidence that the calcium carbonate bodies are located in the periplasm, in invaginations of the cytoplasmic membrane. We describe these invaginations of the cytoplasmic membrane as ‘pockets’ because pockets typically can be accessed from outside and are not closed off from the environment.

## RESULTS

### General Morphology of *Achromatium* Cells

**Figure 1.**
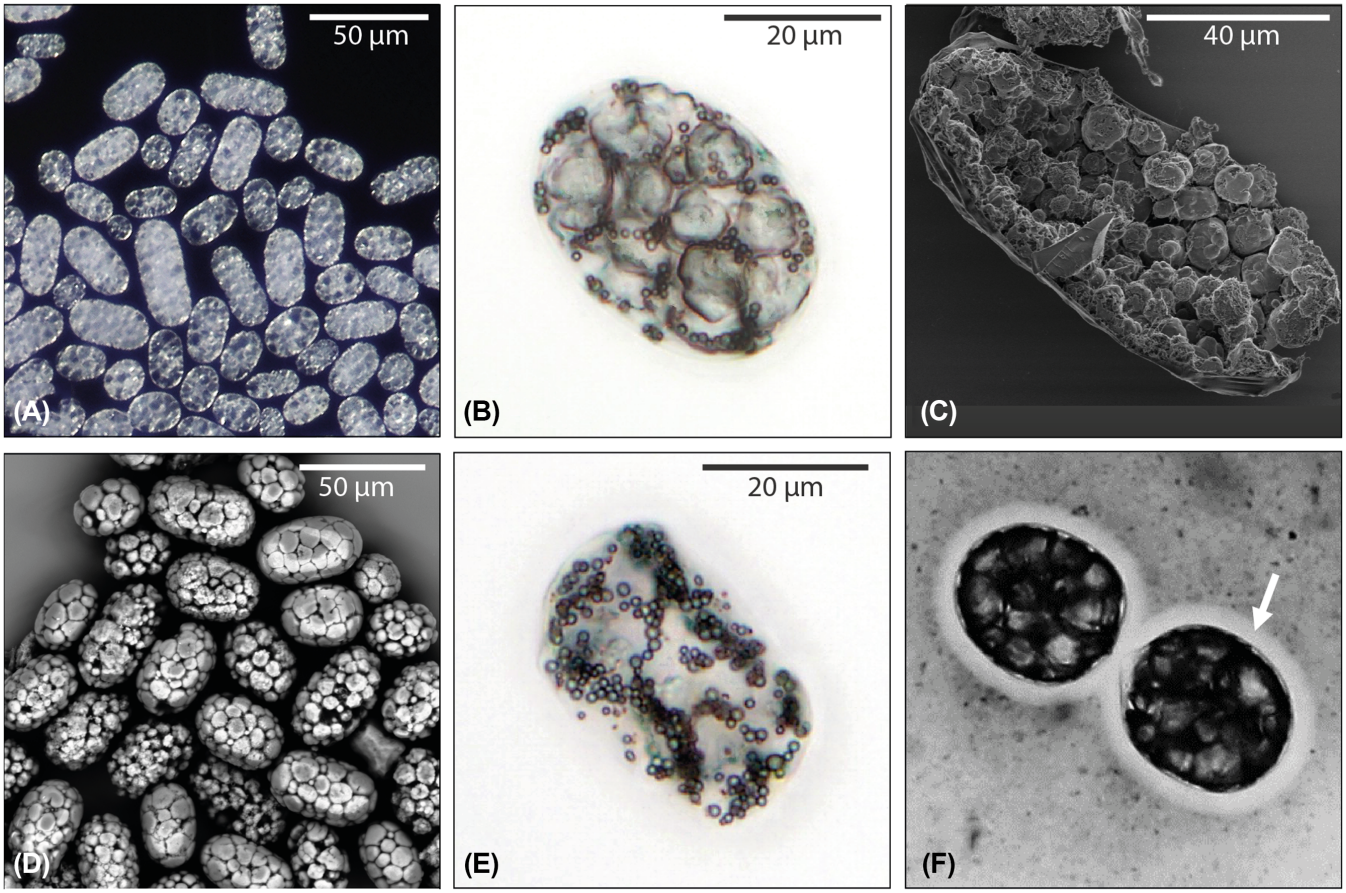
Morphology of *Achromatium* cells. (A) Illumination with incident light gives the cell a white appearance due to multiple reflective inclusions of calcium carbonate and sulfur. (B) Transmitted light reveals individual internal calcium carbonate bodies (large granules) and sulfur globules (small droplets). (C) Scanning electron micrograph of an opened cell showing dense arrangements of calcium carbonate bodies inside the cell. (D) Heterogeneous amounts of internal calcium carbonate bodies, and occasional empty areas between the calcium carbonate bodies of single cells, visualized by scanning electron microscopy with backscattered electrons. (E) *Achromatium* cells in natural populations were regularly calcium carbonate-free, but carried varying amounts of sulfur globules. (F) *Achromatium* cells are often enclosed by slime (arrow pointing to the light, non-stained halo surrounding the cell), visualized by negative staining (Indian ink = black and grey areas).

*Achromatium* cells sampled from the upper sediment layers (0.5 to 1.5 cm) of Lake Stechlin, Germany, showed a broad variability in size (cell length 15 to > 100 µm) and content of calcium carbonate bodies (Figure 1(a)). Most cells were filled with 20 to > 100 calcium carbonate bodies with diameters between 3 and 6 µm (Figure 1(b) and (c)), and numerous smaller sulfur globules (≈1 µm in diameter, Figure 1(b) and (e)). In many cells, individual calcium carbonate bodies were occasionally missing, leaving behind void cavities (Figure 1(d)). Some cells were even entirely free of calcium carbonate (Figure 1(e)). Nevertheless, these cells were motile, and showed the same cell sizes and shapes as their calcium carbonate-filled counterparts. Negative staining with Indian ink showed that many cells were surrounded by slime layers of varying thickness (Figure 1(f)). The slime could be washed off with NaHCO_3_ (50 mmol l^−1^), which also removed the calcium carbonate bodies, but not the sulfur globules from the cells interior. The cells retained their size and shape even when slime layer and calcium carbonate bodies were lost or removed.

### Intracellular Structures

**Figure 2.**
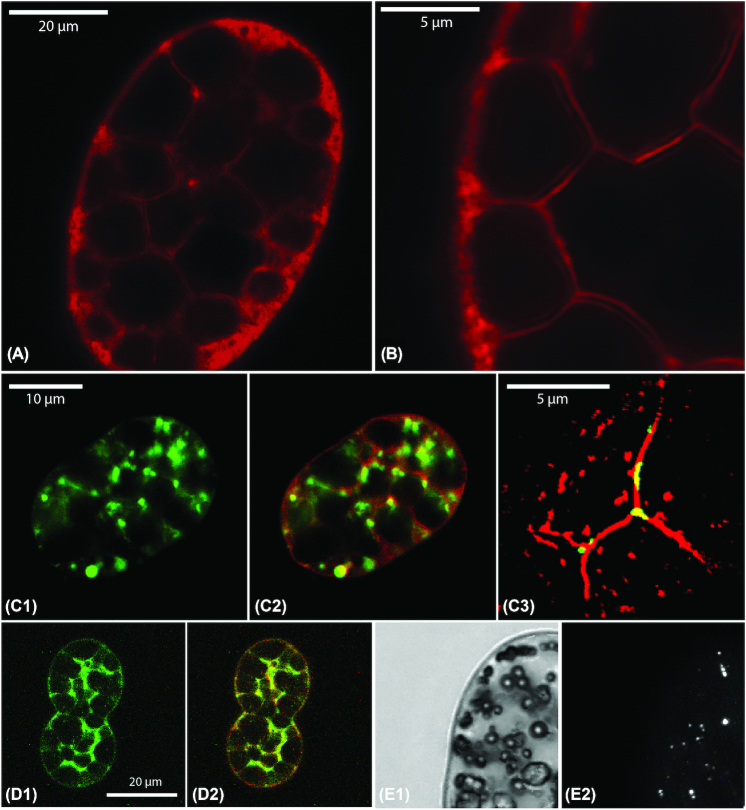
Intracellular structures of *Achromatium*. Nile Red staining visualized membranes at the cell periphery (a) as well as a membrane network stretching in thin threads throughout the cell interior (close-up in b). Nucleic acid staining with SYBR Green I revealed numerous DNA spots (c1) that were co-localized with the membranes as visualized by double staining of SYBR Green I with Nile Red during confocal microscopy (c2) and superresolution microscopy (c3). FITC staining revealed the condensation of the cytoplasm to the interstitial space between the calcium carbonate cavities (d1 and d2, FITC = green signal, Nile Red = red signal). Fura-2 staining of dissolved calcium ions revealed several small spots (white) in the cell interior (e2) that are not co-localized with sulfur granules (spherical inclusions in e1, which is the corresponding transmitted light image of the same field of view as e2).

To study the intracellular cell architecture we used fluorescent dyes, confocal laser-scanning microscopy (CLSM), and superresolution-structured illumination microscopy (SR-SIM). As already shown by Head et al. (2000b) the lipophilic dye Nile Red visualized membranes that surrounded the cell, additionally stretched through the cell interior, and around the calcium carbonate bodies (Figure 2(a) and (b), red signal). The nucleic acid-specific dye SYBR Green I stained multiple DNA spots as shown before for marine (Salman et al. 2015) and freshwater (Head et al. 2000a; Ionescu et al. 2017) *Achromatium* cells (Figure 2(c)). Superresolution imaging after double-staining with SYBR Green I and Nile Red showed that the DNA spots co-localized with the internal membrane regions (Figure 2(c)). FITC (fluorescein isothiocyanate, which binds to amino- and sulfhydryl/or thiol groups of proteins in the cytoplasm) stained thin stretches in the interstitial space between the calcium carbonate cavities (Figure 2(d) and Video 1), confirming that the cytoplasm was condensed to a small volume inside the cells (Salman et al. 2015). By staining with Fura-2, a high-affinity dye for dissolved calcium ions, we observed numerous calcium (Ca^2+^)-enriched spots (Figure 2 (e2)), similar to a previous observation in marine *Achromatium* cells (Salman et al. 2015). These calcium spots were found both in calcium carbonate-filled and calcium carbonate-depleted cells, and did not co-localize with the intracellular sulfur globules (Figure 2 (e1) and 2 (e2)). Their presence was very heterogeneous among the cells, i.e., some cells contained numerous spots whereas others did not stain with Fura-2 at all (Figure S1).

#### Experimentally induced dissolution of calcium carbonate bodies

The dissolution of the calcium carbonate bodies could be induced by different chemical and physical treatments in the laboratory, i.e., with diluted hydrochloric acid, acetic acid, ethanol, ethylenediaminetetraacetic acid (EDTA), sodium bicarbonate, and UV radiation (Table 1). Artificially decalcified cells retained their size and shape. When the suspensions were not acidified, occasionally the precipitation of cubic calcium carbonate crystals (reminiscent of calcite) outside of the cells could be observed (Figure S2). Except for the treatments with acids and UV radiation the cells remained motile. None of the treatments caused a noticeable loss of sulfur globules from the cells.

**Table 1.** Cells showing loss of calcium carbonate bodies upon various treatments

Solution (concentration)		Affected cells after 25 min (%)
Stechlin lake water		3
Hydrochloric acid	5 mM	97
Acetic acid	5 mM	97
Ethanol	5 mM	23
Ethylenediaminetetraacetic acid (EDTA)	5 mM	29
Sodium bicarbonate	5 mM	28
UV radiation	360 nm	31

### Localization of the Calcium Carbonate Bodies

**Figure 3.**
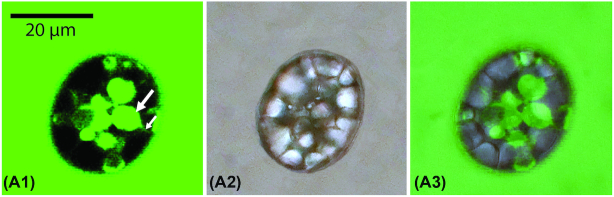
Fluorescein staining. (a) Unfixed *Achromatium* cells with naturally occurring empty cavities were immediately penetrated by the hydrophilic dye fluorescein, and revealed green-fluorescent signals at the same locations, and with reminiscent shapes, as the missing calcium carbonate bodies. (a1) Fluorescein signal in green, big arrow pointing at a shape reminiscent and co-localized with a calcium carbonate cavity, small arrow pointing at a thin green-fluorescent channel-like structure; (a2) transmitted light image of the same cell; (a3) overlay of a1 and a2.

The easy loss of the calcium carbonate bodies from the cells let us hypothesize that these bodies are not completely membrane-enclosed, but are located in extra-cytoplasmic pockets of the periplasm. To test this, we stained *Achromatium* cells with fluorescein, a hydrophilic dye that does not penetrate membranes. If the calcium carbonate bodies were located in invaginations of the cytoplasmic membrane with open connections to the periplasm, we expected penetration of the dye, and the development of fluorescence in the calcium carbonate cavities. Using confocal microscopy, we examined a population of fresh unfixed cells, to which fluorescein had been added, and observed within three minutes that fluorescein had penetrated some of the cells, generating rounded green-fluorescent spots of the typical size and shape of calcium carbonate bodies (Figure 3(a1)). Light microscopy with transmitted light revealed that these cells had naturally occurring empty calcium carbonate cavities at the positions of the green spots (Figure 3(a2)). No fluorescence signal was emitted from cells that were entirely filled with internal calcium carbonate bodies (Figure S3(a)). Control experiments with yeast cells verified that fluorescein did not penetrate membranes of intact cells because also here only the aqueous milieu surrounding the yeast cells was stained (Figure S3(b)).

## DISCUSSION

### Calcium Carbonate Bodies in Extra-Cytoplasmic Pockets

Our study gives a detailed description of the cell architecture of *Achromatium oxaliferum*, in particular of the cellular localization of the calcium carbonate bodies. It was already known (and proven that *Achromatium oxaliferum* does contain carbonate and not oxalate crystals) that acetic acid causes loss of the calcium carbonate bodies (West and Griffiths 1913). In our study, we found various other treatments (diluted HCl, ethanol, bicarbonate and UV radiation) to yield the same result. Different from acetic acid, diluted HCl is not membrane-permeable as it dissolves completely to H^+^ and Cl^−^. The other chemicals tested caused loss of the calcium carbonate bodies at concentrations that should not harm the cells, but point to release of calcium carbonate as a physiological response.

The easy loss of calcium carbonate from the cells (while the sulfur globules were retained), and the rapid entry of a non-membrane-permeable dye into the empty cavities gave proof that the calcium carbonate bodies are not located in the cell's cytoplasm but in extra-cytoplasmic pockets. This finding does not automatically point to a physiological function of the bodies, but might exclude some of those discussed in the literature, e.g., cytoplasmic pH regulation (detailed reviews in Gray 2006; Gray and Head 2014), because protons and other charged molecules cannot pass through membranes by diffusion. Instead, our results re-direct the potential physiological functions of calcium carbonate to the periplasmic space. Buffering capacities in the periplasm could be connected to the formation of polysulfide/sulfur globules in this compartment, which is a process also causing proton fluctuations as known from other sulfur-storing bacteria, e.g., green and purple sulfur bacteria (Pott and Dahl 1998; Schütz et al. 1999; Dahl 2008). The additional still plausible hypothesis based on current evidence is that cells regulate their calcium carbonate body content in order to change buoyancy, and thus migrate vertically in response to sulfide- and electron acceptor availability in the sediment (Babenzien 1991; Gray 2006) or rapidly sink out of the water column back to the sediment after wave-induced resuspension of the upper layer.

### Natural Calcium Carbonate Dynamics

It is known that natural populations of *Achromatium* cells dynamically change the content of calcium carbonate bodies, a process which is possibly correlated with the local concentrations of sulfide and/or oxygen that the cells are exposed to in a natural, dynamic gradient system (Head et al. 2000b; Salman et al. 2015; Yang et al. 2019). We found intracellular spots enriched in dissolved Ca^2+^, as previously described by Salman et al. (2015), however, it remains unclear whether these are located within the calcium carbonate pocket or inside an own cavity. In any case, the finding of condensed spots of dissolved Ca^2+^ ions indicates that the ions are most likely enclosed to prevent diffusion into the cytoplasm. Our imaging experiments showed that the calcium carbonate cavities and the Ca^2+^ spots are located in close proximity, which supports the hypothesis that the dissolved Ca^2+^ ions serve as reservoir and are physiologically connected to the dynamic formation and dissolution of the carbonates (Salman et al. 2015). It has been proposed that *Achromatium* cells must be able to scavenge free calcium ions from the environment, and putatively operate Ca^2+^-pumping ATPases (Head et al. 2000b; Gray 2006). However, the source, fluctuation, and turnover of dissolved calcium used by *Achromatium* remain speculative at this point.

### Cell Shape and Outer Cell Envelope

The outer envelope gives a bacterial cell its shape and stability against osmotic pressure of the cytoplasm. For physical reasons, the required pressure resistance increases with the cell diameter. In previous studies, performing thin sectioning and TEM imaging of subcellular structures, including the cell envelope (de Boer, la Rivière and Schmidt 1971; Head et al. 1996), there was no indication that the cell wall of *Achromatium* is unusually thick. Furthermore, *Achromatium* cells are not entirely filled with cytoplasm, but harbour a network of thin cytoplasmic threads (Figure 2(d); Head et al. 2000b; Salman et al. 2015). Hence, the outer cell envelope does not have to withstand increased osmotic pressure. In support of this is the observation that cells with empty calcium carbonate cavities did not change in size or shape. If the cavities are not cytoplasmic, they are not exposed to the corresponding osmotic pressure.

The slime layers observed in many *Achromatium* cells (Figure 1(f); de Boer, la Rivière and Schmidt 1971; Head et al. 1996) might help to retain the calcium carbonate bodies, but whether they serve as mechanical or chemical barrier remains to be shown. We observed that a short treatment with bicarbonate (50 mM) not only removed the slime layer, but also caused loss of calcium carbonate bodies. The slime layer is probably rich in Ca, which, like other environments rich in extracellular polymeric substances, is complexed by negative residues that bridge the polymers and therefore do not precipitate (Decho and Gutierrez 2017). The bicarbonate buffer we added to the cells probably led to local calcite precipitation, which destabilized these polymer bridges and led to slime dissolution. In absence of a slime layer, calcium carbonate bodies may be directly exposed to a change of the chemical equilibria of carbonate, bicarbonate, and CO_2_, causing calcium carbonate dissolution. Even low bicarbonate concentrations (5 mM, Table 1) caused a slow dissolution of the calcium carbonate bodies, underpinning the connection of chemical equilibria and calcium carbonate stability.

### Cytoplasm Structure, Sulfur Globules and Putative Intracellular Vacuoles

In agreement with previous observations by Head et al. (2000b) and Salman et al. (2015), our localization experiments of DNA, membranes, and cytoplasmic proteins confirmed that the cytoplasm of *Achromatium* is restricted to a thin layer underneath the cell wall and a network of narrow threads spanning between the calcium carbonate cavities. This results in a strong compartmentalization of *Achromatium* cells.

The sulfur globules appear to have a similar distribution as the cytoplasm, but it remains uncertain whether they are cytoplasmic or not. In previous studies (de Boer, la Rivière and Schmidt 1971; Head et al. 1996), thin sectioning and TEM imaging of *Achromatium* did not unravel the specific localization of the sulfur globules. In *Beggiatoa alba*, TEM images have shown that the sulfur globules are located in the periplasm, surrounded by invaginations of the cytoplasmic membrane, and that each sulfur globule is furthermore surrounded by its own type of envelope, possibly a membrane (Strohl et al. 1982). In *Thiothrix*, sulfur globules are suggested to be enclosed by a single-layered membrane and TEM likewise showed their periplasmic location in invaginations of the cytoplasmic membrane (Williams et al. 1987). Among the purple sulfur bacteria, the closest relatives of *Achromatium*, the sulfur globules are also supposedly localized in the periplasm, as they may be coated by proteins with signature sequences indicating export through the cytoplasmic membrane (Pattaragulwanit et al. 1998; Weissgerber et al. 2011). Thus, a similar location would be feasible for the sulfur globules in *Achromatium*. However, we did not detect the corresponding export-indicating signatures in the gene sequences for the coating-proteins in *Achromatium oxaliferum* (own unpubl. work).

A large intracellular vacuole, as known from other large sulfur bacteria, was not detected in our imaging analyses. Anyhow, the presence of the calcium carbonate bodies massively restricts the volume available for a vacuole, meaning that if present at all, vacuoles would be relatively small. In the genomes of brackish (Mansor et al. 2015), freshwater (Ionescu et al. 2017) and marine *Achromatium* (Salman et al. 2016; IMG accession number 2603880209, annotation IDs Ga0065144_11068–15) V-ATPases have been identified. These ATPases are vastly studied in eukaryotes, where they are bound to the membranes of organelles, and function as H^+^/Ca^2+^ antiporter (Schumaker and Sze 1986). They are also occasionally found in bacteria (Mulkidjanian et al. 2007), e.g. in the vacuole-containing *Beggiatoaceae* (Mußmann et al. 2007). The presence of these ATPases could support the argument in favour of the presence of an aqueous, nitrate-containing vacuole. More in line with our findings, though, the functionality of these V-ATPases could be related to the translocation of Ca^2+^ ions between the calcium carbonate-surrounding membrane and the smaller Ca^2+^ ion-containing spots.

In conclusion, the cell body of *Achromatium oxaliferum* is strongly compartmentalized, leading to a massive restriction of cytoplasm but great expansion of the periplasmic space. The incorporation of calcium carbonate into the periplasm of the cell body is a rare phenomenon among bacteria and their possible role has interested researches for decades. Visualization of the unique architecture of these locally highly abundant sulfur-oxidizing microorganisms now lays the foundation for future investigations of the role of calcium carbonate bodies in *Achromatium*.

## MATERIAL AND METHODS

### Sampling Site and Storage of *Achromatium* Cells

Lake Stechlin is an oligo-mesotrophic hardwater lake located near Neuglobsow, Brandenburg, Germany (53°9′5.59″N; 13°1′34.22″E). Freshwater *Achromatium* cells were collected in September 2016 and March 2017 from surface sediments of the shallow shore in front of the Leibniz Institute of Freshwater Ecology and Inland Fisheries. The upper 3 cm of the lake sediment were retrieved with beakers, filled into jars to about a height of 3 cm, and topped with ca. 5 cm of fresh lake water. After return to the lab, the overlying water was reduced to ca. 2 cm, and the jars were stored at 15°C at a 12 h/12 h light/dark cycle. Under these conditions, viable *Achromatium* cells could be maintained for several months.

### Cell Collection and Purification

Prior to each experiment, *Achromatium* cells were freshly collected from the sediment jars. For this, subsamples from the upper centimeter of the sediment were filtered through a mesh with 80 µm pore size to separate *Achromatium* cells from large sand grains and other organisms present in the sediment. The flow-through containing the *Achromatium* cells was collected in a glass petri dish, and was horizontally rotated so that the cells accumulated in the center of the petri dish as previously described (de Boer, la Rivière and Schmidt 1971; Head et al. 1996; Salman et al. 2015; Yang et al. 2019). Tilting of the petri dish and jerky shaking movements resulted in the displacement of organic debris adjacent to the concentrated *Achromatium* cells. The cells were collected with a glass Pasteur pipette and transferred into a fresh petri dish filled with sterile-filtered lake water. The purification steps were repeated until a visibly clean *Achromatium* population was obtained.

### Staining and Fluorescence Microscopy

For staining with fluorescein isothiocyanate (FITC), cells were fixed with 2% formaldehyde for 1 hour at room temperature, washed with sterile lake water, and stained with FITC (0.1 mg ml^−1^) for 1 hour at room temperature in the dark. For staining with SYBR Green I, fresh cells were pipetted onto microscopic slides containing a droplet of the SYBR Green I staining solution, which consisted of 50x SYBR Green I, 380 mM polyvinylalcohol 4–88 (moviol 4–88, Fluka, Switzerland), 70 mM glycerol, 20 µM ascorbic acid, dissolved in 1 ml 1x TRIS-acetate-EDTA (TAE) buffer, and directly imaged. For membrane staining, Nile Red (Sigma-Aldrich, Darmstadt, Germany) was dissolved in DMSO, added to fresh cells in a final concentration of 8 µM, and also immediately imaged. The objective slides for imaging were equipped with an outer lining of tape to hold the cover slip, and to avoid damaging of the large cells through the weight of the cover slip. Stained structures were visualized with a confocal laser scanning microscope (Zeiss LSM 780 with ELYRA PS.1 system) and superresolution-structured illumination microscopy (SR-SIM) using a Plan-Apochromat 63x/1.4 Oil DIC M27 objective and lasers emitting 458 nm (for SYBR Green I and fluorescein) and 514 nm (for Nile Red).

For staining of dissolved calcium ions, Fura-2 AM Calcium Indicator (Molecular Probes, Thermo Fisher Scientific, Bremen, Germany) was dissolved at 1 mM in DMSO and stored at −20°C before and after use. Staining of fresh cells was done in sterile lake water supplemented with 5 µM Fura-2 AM and 0.04% Pluronic (Molecular Probes, Thermo Fisher Scientific, Bremen, Germany) for 1 h in the dark at 20°C before the cells were washed three times in fresh sterile lake water. Stained cells were visualized with a 40x objective on an Olympus IX81 inverse microscope, equipped with an Olympus MT20 Cell-IR Burner and the Olympus filter set U-M2FUR. Z-sectioned images were taken with the Olympus software Xcellence.

### Image Processing

For 3D reconstruction of cellular structures, stacked images were recorded throughout the z-axis of the cell and further processed with PICOLAY (www.picolay.de, Cypionka, Völcker and Rohde 2016). Image processing was done with Zen 3.0 (Zeiss) and PICOLAY.

### Scanning Electron Microscopy

Scanning electron microscopy (SEM) was performed to study the ultrastructure of *Achromatium*. Cells were either fixed in 1% formaldehyde (FA) for 45 minutes at room temperature or processed without fixation. When fixed, FA was carefully removed and the cells were washed four times with sterile lake water. Fixed and unfixed cells were immobilized on silicon wafers, for which they were allowed to settle in a drop of poly-l-lysine solution (0.1 mg ml^−1^) for 5 minutes. Thereafter, excess water was removed, and the cells were stepwise dehydrated in an ethanol series (30%, 50%, 70%, 80% and 96%) for 5 minutes at each step. The cells were then introduced into a critical point dryer (Leica EM CPD300). Finally, the samples were coated with a carbon layer of 2 nm. Samples were imaged with a scanning electron microscope (FEI Quanta 250 FEG) with an Everhart-Thornley secondary electron detector (ETD) and a circular backscatter detector (CBS). For imaging acceleration voltages of 2 and 20 kV were used. We observed that fixation of the cells with FA prior to dehydration caused the formation of holes in the calcium carbonate bodies (Fig. S4). As we did not observe a difference in morphology and shape between fixed and fresh cells we did not fix the cells with FA that are shown in Figure 1.

### Induction of Calcium Carbonate Loss Under Laboratory Conditions

To induce calcium carbonate loss, *Achromatium* cells were incubated with hydrochloric acid, acetic acid, ethanol, ethylenediaminetetraacetic acid (EDTA), sodium bicarbonate (all 5 mM) and UV radiation (Philips TL 5 6 W/08 F6 T5/BLB, 360 nm). The number of cells showing empty calcium carbonate cavities were analyzed on photomicrographs taken through an inverted microscope (Leitz Diavert) at 50-fold magnification.

## Supplementary Material

fiz200_Supplemental_File

## References

[bib1] Babenzien HD. *Achromatium oxaliferum* and its ecological niche. Zentralblatt für Mikrobiologie. 1991;146:41–49.

[bib4] de Boer WE, la Rivière JWM, Schmidt K Some properties of *Achromatium oxaliferum*. Antonie Van Leeuwenhoek. 1971;37:553–63. 4109906 10.1007/BF02218525

[bib2] Cypionka H, Völcker E, Rohde M Erzeugung virtueller 3D-Bilder mit jedem Lichtmikroskop oder REM. BIOspektrum. 2016;22:143–45.

[bib3] Dahl C. Inorganic sulfur compounds as electron donors in purple sulfur bacteria, In: Hell R. Dahl C. Knaff D. Leustek T.(ed.). Sulfur Metabolism in Phototrophic Organisms. Dordrecht, The Netherlands: Springer 2008, 289–317.

[bib5] Decho AW, Gutierrez T Microbial Extracellular Polymeric Substances (EPSs) in Ocean Systems. Front Microbiol. 2017;8:922. 28603518 10.3389/fmicb.2017.00922 PMC5445292

[bib6] Glöckner FO, Babenzien HD, Wulf J et al. Phylogeny and Diversity of *Achromatium oxaliferum*. Syst Appl Microbiol. 1999;22:28–38. 10188276 10.1016/S0723-2020(99)80025-3

[bib8] Gray ND, Head IM The family *Achromatiaceae*. In: Rosenberg Eugeneet al. (eds.). The Prokaryotes: Gammaproteobacteria. Berlin, Heidelberg: Springer Berlin 2014, 1–14.

[bib9] Gray ND, Howarth R, Rowan A et al. Natural communities of *Achromatium oxaliferum* comprise genetically, morphologically, and Eeologically distinct Ssbpopulations. Appl Environ Microbiol. 1999;65:5089–99. 10543827 10.1128/aem.65.11.5089-5099.1999 PMC91685

[bib7] Gray ND. The unique role of intracellular calcification in the genus *Achromatium*. In: Shively Jessup M.(ed.). Inclusions in Prokaryotes. Berlin, Heidelberg: Springer Berlin 2006, 299–309.

[bib10] Head IM, Gray ND, Babenzien H et al. Uncultured giant sulfur bacteria of the genus *Achromatium*. FEMS Microbiol Ecol. 2000a;33:171–80. 11098068 10.1111/j.1574-6941.2000.tb00739.x

[bib11] Head IM, Gray ND, Clarke KJ et al. The phylogenetic position and ultrastructure of the uncultured bacterium *Achromatium oxaliferum*. Microbiology. 1996;142:2341–54. 8828202 10.1099/00221287-142-9-2341

[bib12] Head IM, Gray ND, Howarth R et al. *Achromatium oxaliferum*: Understanding the unmistakable. Adv Microb Ecol. 2000b;16:1–40.

[bib13] Ionescu D, Bizic-Ionescu M, De Maio N et al. Community-like genome in single cells of the sulfur bacterium *Achromatium oxaliferum*. Nat Commun. 2017;8:455. 28878209 10.1038/s41467-017-00342-9 PMC5587575

[bib14] Lane N, Martin W The energetics of genome complexity. Nature. 2010;467:929. 20962839 10.1038/nature09486

[bib15] La Rivière JWM, Schmidt K Morphologically conspicuous sulfur-oxidizing eubacteria. The Prokaryotes: A Handbook on Habitats, Isolation, and Identification of Bacteria. 1981;1037–48.

[bib16] Larkin JM, Henk MC Filamentous sulfide-oxidizing bacteria at hydrocarbon seeps of the Gulf of Mexico. Microsc Res Tech. 1996;33:23–31. 8820662 10.1002/(SICI)1097-0029(199601)33:1<23::AID-JEMT4>3.0.CO;2-1

[bib17] Mansor M, Hamilton TL, Fantle MS et al. Metabolic diversity and ecological niches of *Achromatium* populations revealed with single-cell genomic sequencing. Front Microbiol. 2015;6:822. 26322031 10.3389/fmicb.2015.00822 PMC4530308

[bib18] Mulkidjanian AY, Makarova KS, Galperin MY et al. Inventing the dynamo machine: the evolution of the F-type and V-type ATPases. Nat Rev Microbiol. 2007;5:892. 17938630 10.1038/nrmicro1767

[bib19] Mußmann M, Hu FZ, Richter M et al. Insights into the genome of large sulfur bacteria revealed by analysis of single filaments. PLoS Biol. 2007;5:e230. 17760503 10.1371/journal.pbio.0050230 PMC1951784

[bib20] Pattaragulwanit K, Brune DC, Trüper HG et al. Molecular genetic evidence for extracytoplasmic localization of sulfur globules in *Chromatium vinosum*. Arch Microbiol. 1998;169:434–44. 9560425 10.1007/s002030050594

[bib31] Pott S, Dahl C Sirohaem sulfite reductase and other proteins encoded by genes at the dsr locus of *Chromatium vinosum* are involved in the oxidation of intracellular sulfur. Microbiology. 1998;144:1881–94. 9695921 10.1099/00221287-144-7-1881

[bib21] Salman-Carvalho V, Fadeev E, Joye SB et al. How clonal is clonal? Genome plasticity across multicellular segments of a “*Candidatus* Marithrix sp.” filament from sulfidic, riny seafloor sediments in the Gulf of Mexico. Front Microbiol. 2016;7:1173. 27536274 10.3389/fmicb.2016.01173 PMC4971068

[bib23] Salman V, Berben T, Bowers RM et al. Insights into the single cell draft genome of “*Candidatus* Achromatium palustre”. Stand Genomic Sci. 2016;11:28. 27014417 10.1186/s40793-016-0146-x PMC4806510

[bib22] Salman V, Yang T, Berben T et al. Calcium carbonate-accumulating large sulfur bacteria of the genus *Achromatium* in Sippewissett Salt Marsh. ISME J. 2015;9:2503–14. 25909974 10.1038/ismej.2015.62 PMC4611513

[bib24] Schewiakoff W Über einen neuen bakterienähnlichen Organismus des Süßwassers. Habilitationsschrift, Heidelberg, Germany 1893.

[bib25] Schorn S, Cypionka H A crispy diet: grazers of *Achromatium oxaliferum* in Lake Stechlin sediments. Microb Ecol. 2018;76:584–87. 29492594 10.1007/s00248-018-1158-4 PMC6132539

[bib26] Schulz HN, Brinkhoff T, Ferdelman TG et al. Dense populations of a giant sulfur bacterium in Namibian shelf sediments. Science. 1999;284:493–95. 10205058 10.1126/science.284.5413.493

[bib27] Schulz HN, Jørgensen BB Big bacteria. Annu Rev Microbiol. 2001;55:105–37. 11544351 10.1146/annurev.micro.55.1.105

[bib28] Schumaker KS, Sze H Calcium transport into the vacuole of oat roots. Characterization of H^+^/Ca^2+^ exchange activity. J Biol Chem. 1986;261:12172–78. 2427517

[bib29] Schütz M, Maldener I, Griesbeck C et al. Sulfide-quinone reductase from *Rhodobacter capsulatus*: requirement for growth, periplasmic localization, and extension of gene sequence analysis. J Bacteriol. 1999;181:6516–23. 10515944 10.1128/jb.181.20.6516-6523.1999 PMC103789

[bib30] Strohl WR, Howard KS, Larkin JM Ultrastructure of *Beggiatoa alba* Strain B15LD. Microbiology. 1982;128:73–84.

[bib32] Weissgerber T, Zigann R, Bruce D et al. Complete genome sequence of *Allochromatium vinosum* DSM 180T. Stand Genomic Sci. 2011;5:311–30. 22675582 10.4056/sigs.2335270 PMC3368242

[bib33] West GS, Griffiths BM The lime-sulphur bacteria of the genus *Hillhousia*. Ann Bot (Lond). 1913;27:83–91.

[bib34] Williams TM, Unz RF, Doman JT Ultrastructure of *Thiothrix* spp. and “Type 021N” Bacteria. Appl Environ Microbiol. 1987;53:1560–70. 16347385 10.1128/aem.53.7.1560-1570.1987 PMC203910

[bib35] Yang T, Teske A, Ambrose W et al. Intracellular calcium carbonate and sulfur dynamics of *Achromatium* cells observed in a lab-based enrichment and aerobic incubation experiment. Antonie Van Leeuwenhoek. 2019;112:263–74. 30194507 10.1007/s10482-018-1153-2

