# Supplementary material for: Cell Architecture of the Giant Sulfur Bacterium Achromatium oxaliferum: Extra-cytoplasmic Localization of Calcium Carbonate Bodies
Source: FEMS Microbiol Ecol. 2019 Dec 24;96(2):fiz200. doi: 10.1093/femsec/fiz200 (PMC6995342; doi:10.1093/femsec/fiz200)
Supplement: fiz200_Supplemental_File [file fiz200_supplemental_file.docx]

**Supplementary Figures**


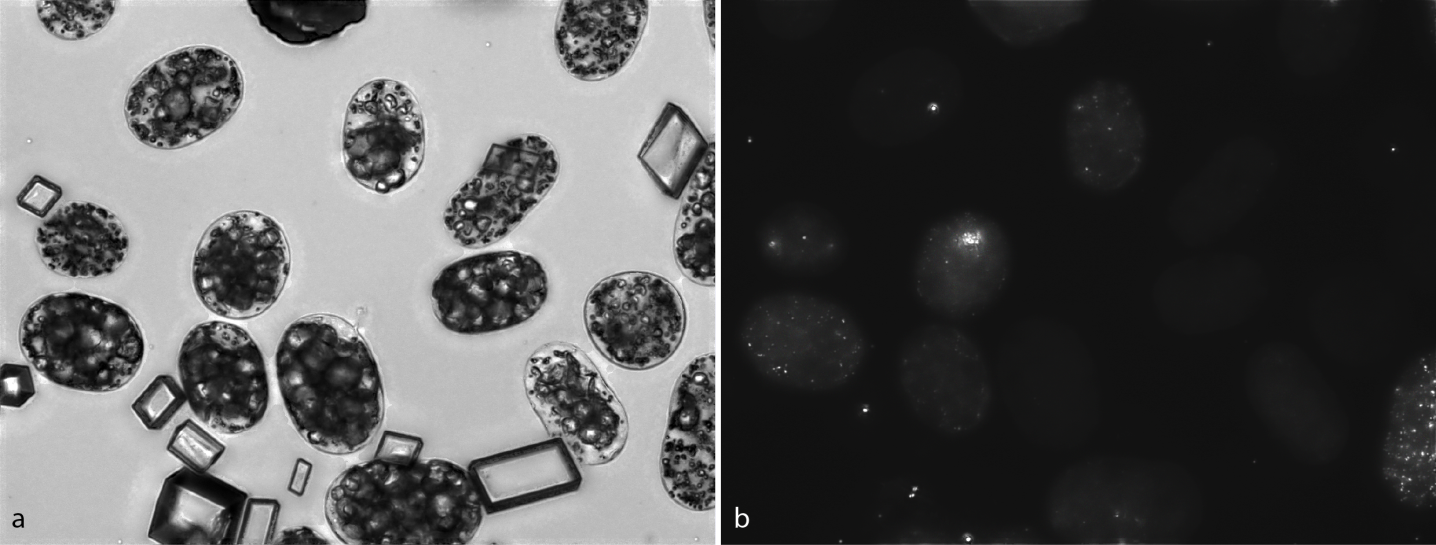


Figure S1. Heterogeneous distribution of spots enriched in dissolved calcium ions in *Achromatium* cells. A population of *Achromatium* cells with varying content of calcium carbonate bodies per cell (a) showed heterogeneous amounts of dissolved Ca^2+^ inside their cells (b; white signals).


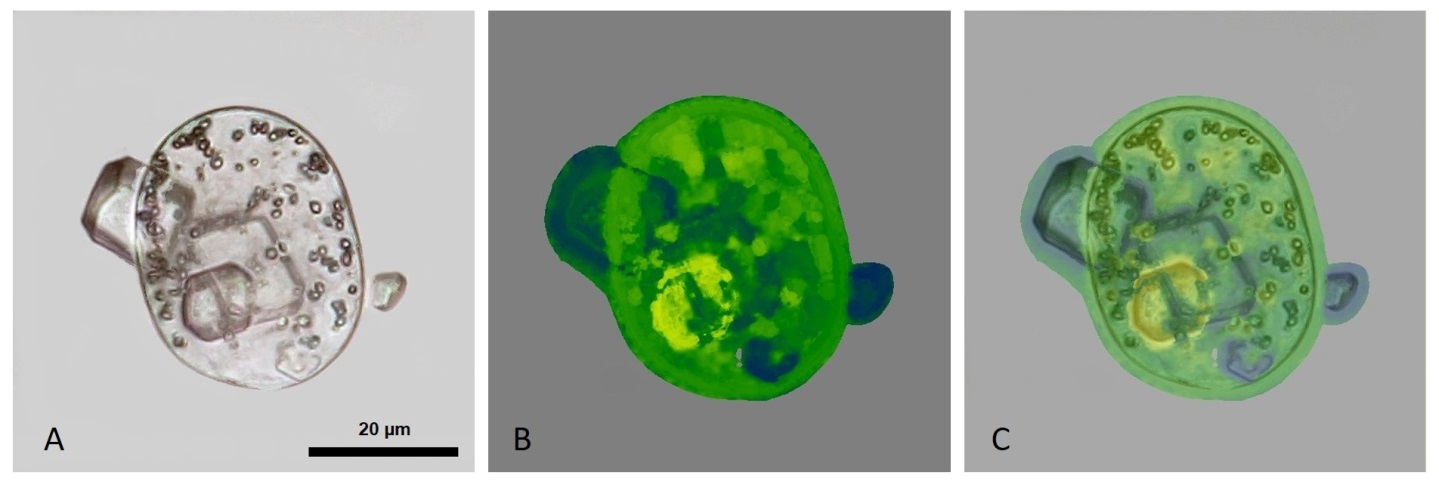


Figure S2. Precipitation of external crystalline calcium carbonate after UV radiation of an individual *Achromatium* cell. A cell harboring calcium carbonate bodies was exposed for 10 min to the light of the 50 W mercury vapor lamp of a Zeiss Axioscope without filter. After 20 min, pictures were taken. (A) Stacked image generated by focus stacking of 34 images with PICOLAY (www.picolay.de). (B) PICOLAY depth map from yellow (top) over green (middle) to blue (bottom). (C) Overlay of A and B showing that external crystals were deposited on top or underneath the cell, while the sulfur globules were still inside.


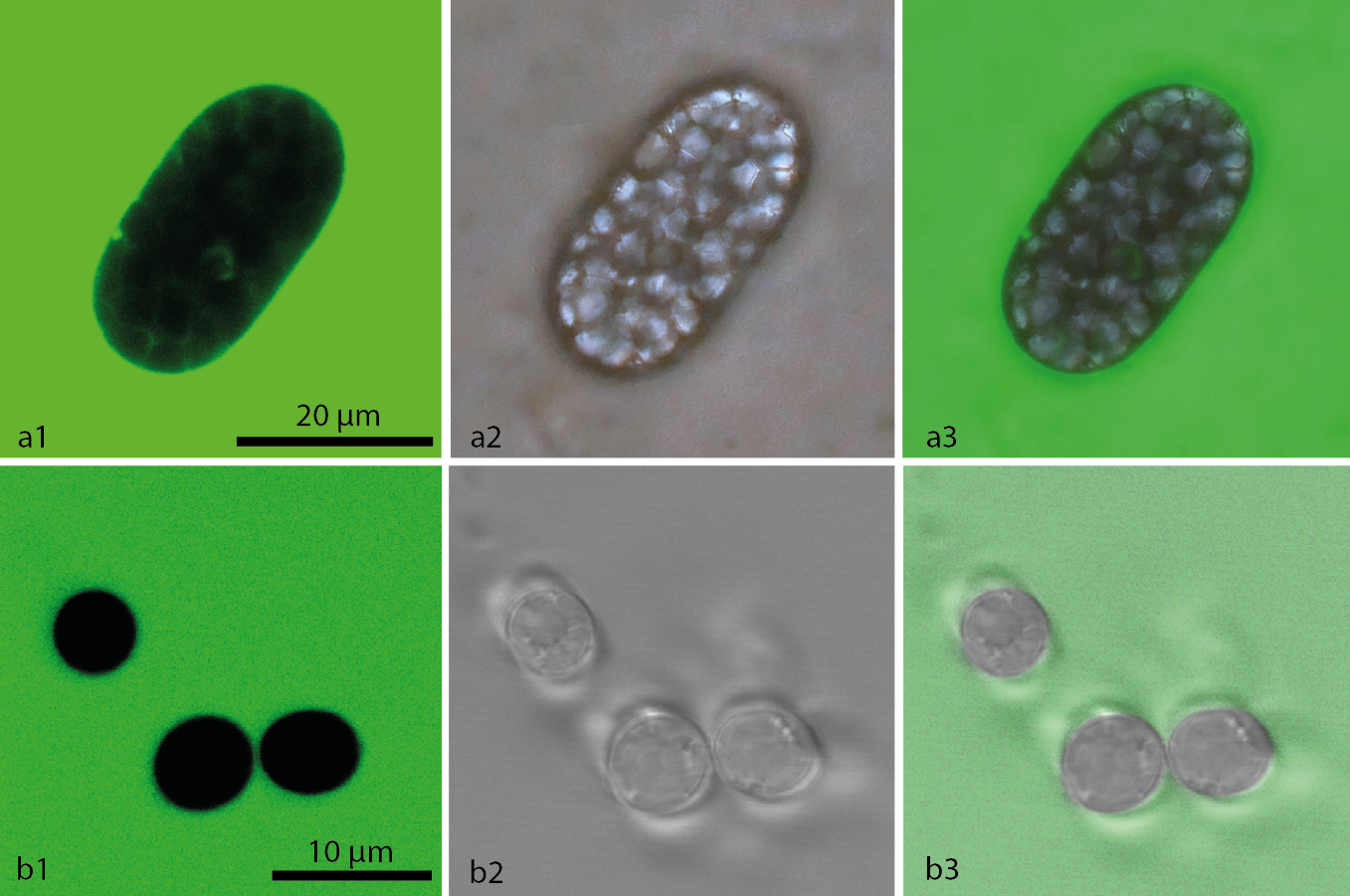


Figure S3. Fluorescein staining. (a) *Achromatium* cells entirely filled with calcium carbonate bodies were not penetrated by fluorescein (a1: fluorescein signal in green; a2: transmitted light image; a3: overlay of a1 and a2). (b) Control staining with yeast cells, confirming that intact membranes are not penetrated by fluorescein (b1: fluorescein signal in green; b2 transmitted light image; (b3) overlay of b1 and b2).


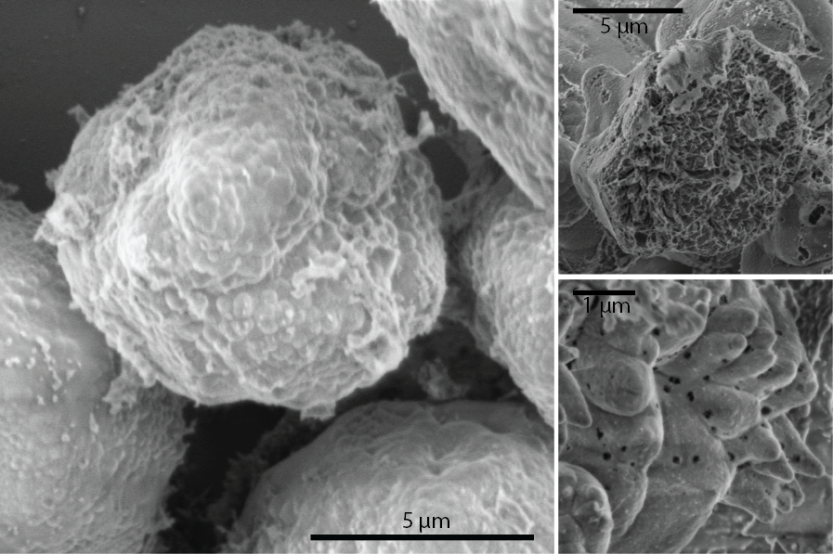


Figure S4. Effect of formaldehyde treatment on calcium carbonate bodies in *Achromatium* cells. (a) In fresh cells we observed the typical morphology of calcium carbonate bodies when viewed with SEM. (b) In FA-fixed cells we observed that the calcium carbonate bodies were partly dissolved and often showed small holes in their structures (c).
